# Analysis of tear film spatial instability for pediatric myopia under treatment

**DOI:** 10.1038/s41598-020-71710-7

**Published:** 2020-09-08

**Authors:** Wan-Hua Cho, Po-Chiung Fang, Hun-Ju Yu, Pei-Wen Lin, Hsiu-Mei Huang, Ming-Tse Kuo

**Affiliations:** Department of Ophthalmology, Kaohsiung Chang Gung Memorial Hospital and Chang Gung University College of Medicine, Kaohsiung, Taiwan

**Keywords:** Biological techniques, Health care, Medical research, Signs and symptoms

## Abstract

In Taiwan, the prevalence of myopia in children between 6 and 18 years old is over 80%, and high myopia accounts for over 20%, which turned out to be in the leading place worldwide. Orthokeratology and low-dose atropine are proven treatments to reduce myopia progression, though the potential corneal disturbances remain an issue in young populations. The alteration of the tear film is widely discussed but there is no consensus to date, so we aim to investigate the tear film spatial instability in children with myopia control using atropine or orthokeratology. Thirty-eight treatment-naïve participants and 126 myopic children under treatments were enrolled. The ocular surface homeostasis, spatial distribution of tear break-up, and high-order aberrations (HOAs) of the corneal surface were assessed. We found out that myopic children treated with either atropine or orthokeratology showed ocular surface homeostasis similar to that in treatment-naïve children. Nevertheless, children treated with orthokeratology presented higher HOAs (*p* < 0.00001) and a tendency of the first tear break-up zone at the inner half of the cornea (*p* = 0.04). This unique spatial instability of the tear film associated with myopia treatment might provide a more focused way of monitoring the pediatric tear film instability.

## Introduction

Many studies have revealed differences in the prevalence of myopia across different regions and ethnicities, and the increased rate of myopia is most prominent in Asian/Pacific children^1,2^. Current consensus on lifestyle modifications for myopia prevention includes increasing the duration of outdoor activities and decreasing the duration of near-work activities^3–5^. On the other hand, clinical methods for retarding myopia progression after its onset include different concentrations of atropine and wearing the orthokeratology lens overnight^6–9^. However, adverse effects associated with cycloplegics include photophobia, accommodation losses, allergic conjunctivitis, and dermatitis^10^. Adverse effects of orthokeratology include corneal staining and microbial keratitis that may be attributed to inadequate tear film homeostasis^11^. Microbial keratitis is a rare but unneglectable vision-threatening complication with an estimated incidence rate of 7.7 cases per 10,000 patient years, similar in extended or overnight lens wear^11,14,30^, which can be prevented by a normal tear film. Therefore, the concern over tear film instability in children is increasing and was announced recently, especially for myopic children under orthokeratology treatment^12–15^.

Potential causes of contact lens-related discomfort and tear film instability include increased evaporation, thinning of the pre-lens tear film, and incomplete blinking^16^. However, these traditional concerns are not applicable in orthokeratology, since it is worn overnight^15,16^. Wearing the orthokeratology lens is thought to alter the ocular surface and induce tear film instability, potentially causing severe ocular surface complications in myopic children under treatment^17–19^. The possible mechanisms associated with tear film instability may include thinning of the tear film lipid layer as a result of the loss of meibomian glands based on contact-lens-induced aggregation of desquamated epithelial cells into keratotic clusters that block the meibomian duct^12,17,19^, conjunctival fold and metaplasia, reduction in goblet cell density, lid wiper epitheliopathy^18^, and upregulation of proinflammatory mediators induced by tissue reshaping^20^. However, no consensus has been reached till now.

Spatial differences in tear film stability have also been identified in a variety of ocular surface diseases. For instance, patients with aqueous tear-deficient dry eyes are reported to be more likely to have initial tear film break-ups in the inferior nasal quadrant, and corneal fluorescein staining, at the same time, commonly appears as interpalpebral or inferior staining, supporting the clinical features of dry eye^21^. The above findings may be explained by the association between decreased noninvasive keratograph break up times in inferior peripheral locations and the related lower lipid layer thicknesses^22^. On the other hand, initial tear film break-ups in patients with cataracts and dry eye syndrome are located at the inferior periphery and the superior central cornea^23^. Different break-up patterns may also reflect different pathophysiologies of dry eye^24^. Understanding the spatial differences in tear film stability offers an additional evaluation of the ocular surface and provides effective preoperative evaluation of tear film break-up regularity to avoid postoperative aggravation of dry eye syndrome^21–24^. These spatial differences in tear film stability may not only provide explanations regarding the disease entity but also act as potential hallmarks of ocular surface diseases. Therefore, we aimed to investigate the presence of tear film spatial instability in myopic children under different treatment modalities. We also aimed for early detection of tear film alteration for preventing further damage from any possible transient ocular surface injuries that may be associated with myopia treatments.

## Material and methods

### Participants

The present prospective cross-sectional study was conducted for investigating and monitoring the tear film stability in children under stable myopia control at the Pediatric Ophthalmology department of Kaohsiung Chang Gung Memorial Hospital between December 2017 and May 2018. All procedures involving human participants followed the tenets of the Declaration of Helsinki. Ethics committee approval was obtained from Chang Gung Medical Foundation Institutional Review Board (No. 201701393B0, October 6, 2017). All participants and their parents were clearly informed about the aim and procedure of this study and the informed consent was obtained from the parents/legally authorized representatives of participants.

We included treatment-naïve and myopic children aged 6–15 years under stable and habitual myopia control. Ocular history and findings of slit-lamp examination of each participant were carefully assessed by senior pediatric ophthalmologists. Participants with previous eyelid and ocular surgeries and current or recent ocular infection or inflammation within 6 months were excluded. The subjects were classified into the normal control group, the atropine group, the orthokeratology group, and the combined (atropine and orthokeratology) group. The right eye of each subject was used for assessment of general tear film characteristics, tear film break-up morphology, and high-order aberrations (HOAs).

### Assessment protocol

Each subject underwent evaluation procedures in the same order after general instructions by a masked examiner. The right eye of each subject was assessed for ocular surface homeostasis and HOAs of the cornea in the following order by masked examiners: tear meniscus height (TMH), corneal surface aberrometry, ocular surface redness scan (R-scan), and non-invasive tear film break-up time (TBUT).

### Selected ocular surface homeostasis markers and HOAs

For elucidating the tear film stability in children, Keratograph 5 M (K5M, OCULUS Optikgeräte GmbH, Wetzlar, Germany), a Placido-based and non-invasive tear film analyzer equipped with corneal surface aberrometry, was adopted to obtain the information regarding the tear film stability, potentially associated homeostatic markers, and aberrometry indices of the ocular surface. Three critical classes of ocular surface homeostasis markers were analyzed in this study including dynamic tear film stability, tear secretion amount, and severity of ocular surface inflammation.

#### Evaluation of dynamic tear film stability

With 880 nm ring illumination to prevent glare during assessment of non-invasive TBUT with video keratoscopy using the tear film analyzer^25^, a series of parameters associated with dynamic tear film stability were obtained from each subject. All participants were instructed to look straight ahead, blink twice, and then try their best not to blink as long as they could. Four indices were generated by automatic detection and calculation after completing the examination: (1) **first TBUT (f-TBUT)**, the time at which the first distortion in the reflected Placido ring occurs; (2) **average TBUT (a-TBUT)**, related to the localized TBUTs and calculated based on the average time of all detected perturbations; (3) **measure time**, the duration between the second blink that initiated the recording and the last complete blink; and (4) **classification**, performed automatically on a scale of 1–4 according to increasing severity.

The K5M marked 24 equal portions (each of 15°), and 8 concentric circles within the corneal surface (each circle an additional 1 mm away from the center). With the recorded videos, we proposed 5 parameters that could reflect the tear film stability more deeply: (1) **number of first break-up areas** (based on total 192 areas), (2) **number of first break-up quadrants** (based on total 4 quadrants: superior-temporal/superior-nasal/inferior-temporal/inferior-nasal), (3) **number of final break-up areas**, (4) **number of final break-up quadrants**, and (5) **break-up slope**, reflecting the break-up speed.

$$\text{break-up slope}= {A}_{f}\div ({T}_{m}- {T}_{f})$$

where *f* designates the first break-up, *m* corresponds to the total measurements, *A* indicates the numbers of areas, and *T* stands for time.

**Figure 1:**
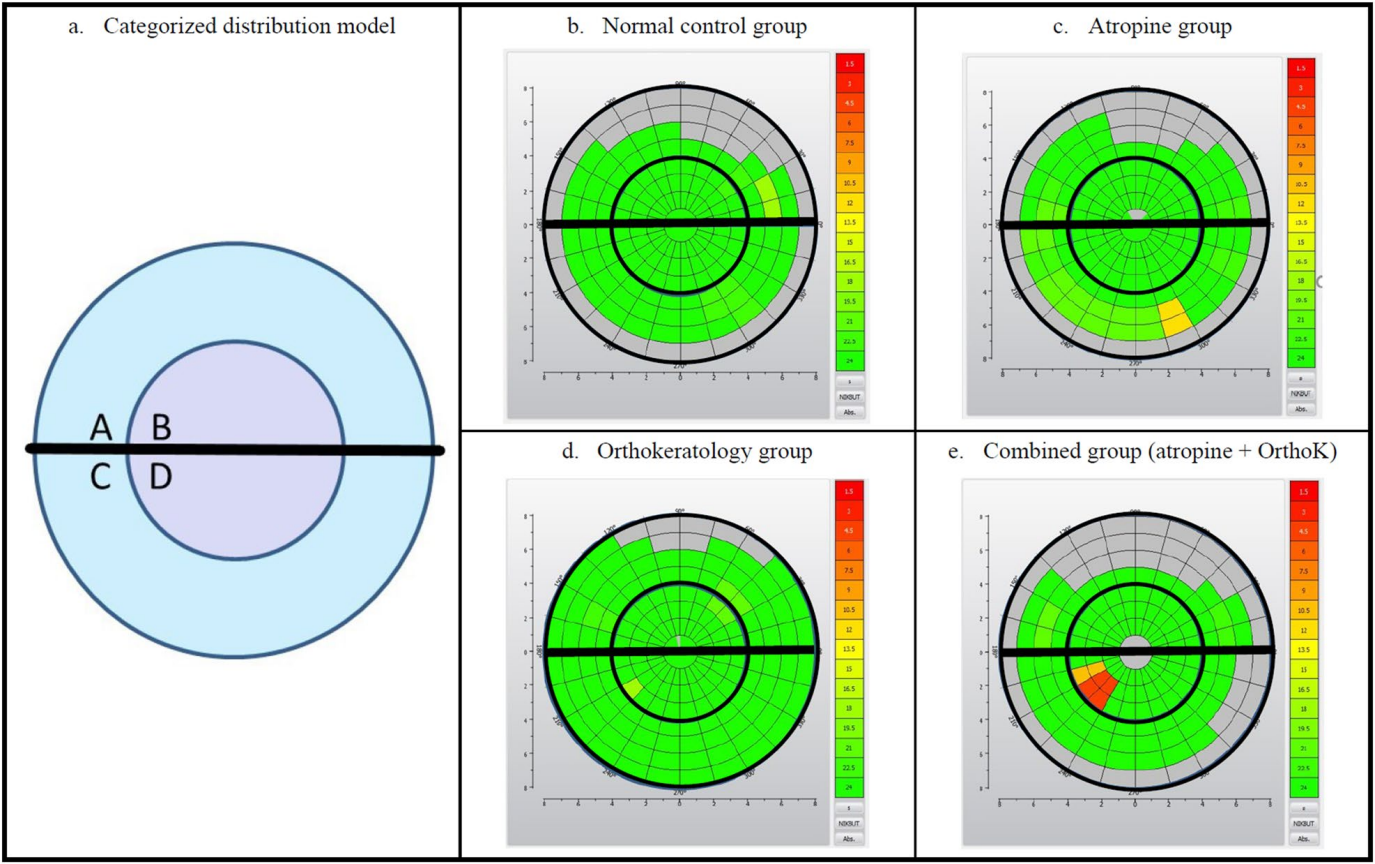
Categorized distribution model and representative break-up-time-based color-coded maps. The first break-up area on the cornea was divided to zones A to D according to the distribution on the superior/inferior half, the inner (less than 4 mm from the center)/outer (more than 4 mm from the center) half. Sub-figures (**b**–**e**) represents typical characteristics of tear break-up performance.

Moreover, we newly categorized the first break-up locations into zones A to D according to the distribution on the superior or the inferior half and the outer (more than 4 mm from the center) or the inner (less than 4 mm from the center) half (Fig. 1). Each area on the color-coded map was indicated with different colors according to its f-TBUT.

#### Quantification of the amount of tear secretion

All subjects underwent imaging with the K5M tear film analyzer with illumination using 4 infrared diodes of 880 nm wavelength^26^. To ensure a dark background for better assessment, the white ring illumination used on the corneal aberrometry was turned down. Central TMH was measured from the curve of the lower lid margin to the top of the tear lake along the vertical line extended from the corneal center with an integrated ruler^27^. The images were captured thrice and the mean values were recorded after each blink for each subject.

#### Quantification of the severity of ocular surface inflammation

The light source was shifted to white ring illumination on the same tear film analyzer for R-scan evaluation^28,29^. All participants were directed to fixate and focus on the mark inside the camera after blinking and the 22-mire Placido ring system would reflect on the entire corneal area. Thus, the R-scan could detect the blood vessels of the conjunctiva and quantify the severity of redness using a built-in software calculator. The index of mean bulbar redness score was obtained within 10 s.

#### Measuring HOAs of the ocular surface

HOAs of the ocular surface represent ocular surface irregularities caused by the anterior corneal surface and the tear film. With the built-in software and pupil size 6 mm, the data were analyzed quantitatively with Fourier analysis and Zernike analysis, which expands the set of Zernike polynomials up to the sixth order. The root mean square (RMS) was calculated to represent the wavefront aberrations. A higher aberration coefficient indicated a decline in the optical quality.

### Statistical analysis

All statistical analyses were performed using IBM SPSS statistics version 22 (IBM Corp., Armonk, NY, USA) and Microsoft Excel 2010 (Microsoft Corporation, Redmond, WA, USA). Pearson’s correlation coefficient was used to analyze the correlations between HOAs and parameters of dynamic tear film stability. Analysis of variance (ANOVA) with Fisher's least significant difference (LSD) post hoc test, Student's *t *test, Chi-squared test, Kruskal–Wallis test, and Fisher’s exact test were used to test the statistical differences in these target parameters among patient groups under different myopia-control treatments. Age-adjusted *p *value was analyzed using multivariate analysis of covariance through stepwise model selection of a multiple regression model adjusted by controlling the confounding variable of age. Statistical significance was set at *p* < 0.05.

### Ethical approval

All procedures involving human subjects adhered to the Declaration of Helsinki. Institutional Review Board (IRB)/Ethics Committee approval was obtained from the Committee of Medical Ethics and Human Experiments of Chang Gung Memorial Hospital (CGMH, Taiwan).

### Meeting presentation

A part of our result has been presented in the 59th Annual Meeting of the Ophthalmological Society of Taiwan in November 2018.

### Informed consent

Informed consent was obtained from the parents/legally authorized representatives of participants.

## Results

### Participants

Thirty-eight healthy participants (18 male and 20 female), 40 patients under myopia control with atropine (21 male and 19 female), 44 patients under myopia control with orthokeratology (21 male and 23 female), and 42 patients under myopia control with both atropine and orthokeratology (15 male and 27 female) were enrolled (Table 1). There was a significant age difference among the groups (9.39 ± 2.67, 11.23 ± 2.36, 12.23 ± 1.71, and 12.19 ± 1.60 years, respectively, *p* < 0.0001). However, there was no significant sex difference among the groups.

**Table 1:** Demographic data of the study participants for tear film dynamic evaluations.

	Normal control group (n = 38)	Atropine group (n = 40)	Orthokeratology group (n = 44)	Combined group (atropine + OrthoK) (n = 42)	*p* value	Age-adjusted *p* value^i^
Age (years)	9.39 ± 2.67	11.23 ± 2.36	12.23 ± 1.71	12.19 ± 1.60	< 0.0001^g^	–
Gender ratio (M/F)	18/20	21/19	21/23	15/27	0.58^h^	0.494
TMH (mm)	0.24 ± 0.05	0.25 ± 0.07	0.24 ± 0.06	0.25 ± 0.09	0.94^g^	0.743
Bulbar redness score	0.64 ± 0.23	0.62 ± 0.38	0.62 ± 0.23	0.60 ± 0.23	0.27^g^	0.176
f-TBUT (s)^a^	11.04 ± 6.88	9.45 ± 6.69	10.57 ± 7.64	9.52 ± 6.40	0.70^g^	0.468
a-TBUT (s)^b^	13.99 ± 7.00	11.45 ± 6.60	13.22 ± 7.29	13.37 ± 6.01	0.40^g^	0.275
Measure time (s)	18.40 ± 7.45	15.32 ± 7.10	17.86 ± 8.00	18.79 ± 6.69	0.14^g^	0.126
Classification	0 (0–1)	1 (0–2)	1 (0–2)	1 (0–1)	0.72^g^	0.232
First-TBU areas^c^	2 (2–3)	2 (2–4)	2 (1.25–3.75)	2 (1.75–4)	0.91^g^	0.833
First-TBU quadrants^d^	1 (1–1)	1 (1–1)	1 (1–1)	1 (1–1)	0.94^g^	0.882
Final TBU areas^e^	9 (4–14)	8 (2–16)	13 (4–21.75)	12.5 (7–21)	0.23^g^	0.340
Final-TBU quadrants^f^	2 (1–3)	2 (1–3)	2 (1–3.75)	2 (2–3)	0.70^g^	0.231
Break-up slope (1/s)	2.40 ± 3.02	2.77 ± 2.60	3.06 ± 4.21	2.29 ± 2.68	0.53^g^	0.674

Parameters were shown by mean ± SD, numbers/numbers, or median (25% quartile–75% quartile) according to the character of each parameter. *p* < 0.05 was recognized as statistical difference.

^a^f-TBUT, the first tear break-up time.

^b^a-TBUT, the average tear break-up time.

^c^First-TBU areas, the numbers of the first tear break-up areas.

^d^First-TBU quadrants, numbers of the first breakup quadrants.

^e^Final TBU areas, numbers of the final breakup areas.

^f^Final-TBU quadrants, numbers of the final breakup quadrants.

^g^Kruskal–Wallis test statistics.

^h^Fisher exact test were used to test the group difference.

^i^Age-adjusted *p* value was analyzed by multivariate analysis of covariance through stepwise model selection of a multiple regression model that was adjusted by controlling the confounding variable of the age.

### Ocular surface presentations

There were no significant differences in parameters representing the dynamic tear film stability (f-TBUT, a-TBUT, measure time, classification, number of first break-up areas, number of first break-up quadrants, number of final break-up areas, number of final break-up quadrants, and break-up slope), the amount of tear secretion (TMH), and the severity of ocular surface inflammation (bulbar redness score) among the groups.

However, in children under myopia control with atropine, participants who underwent a longer treatment duration (more than 2 years) presented better dynamic tear film stability than those who underwent a shorter treatment duration (less than 2 years) (f-TBUT: 11.10 ± 7.09 vs. 6.75 ± 5.14 s, *p* = 0.03; a-TBUT: 13.20 ± 6.58, 8.57 ± 5.77 s, *p* = 0.02). Similarly, in children under myopia control with orthokeratology, participants who underwent a longer treatment duration (more than 3 years) presented better dynamic tear film stability than those who underwent a shorter treatment duration (less than 3 years) (f-TBUT: 15.80 ± 8.30 vs. 8.61 ± 6.48 s, *p* = 0.002; a-TBUT: 17.40 ± 6.95, 11.65 ± 6.88 s, *p* = 0.009). In contrary, TMH and bulbar redness score was not affected by the treatment duration.

### HOAs in children under different myopia treatments

The age-adjusted analysis revealed that children under myopia control with orthokeratology had significantly higher HOAs (Fourier analysis and Zernike analysis) than children without orthokeratology (*p* < 0.00001) (Table 2). There was no significant correlation between HOAs and parameters representing the dynamic tear film stability among the 4 groups. In myopic children treated with orthokeratology, the high HOA group (irregularity > 0.05 RMS or aberration coefficient > 2.5 μm) showed longer measure times than the low HOA group, but with a low power of significance (*p* = 0.068 and 0.088 for irregularity and aberration coefficient, respectively).

**Table 2:** High-order aberrations (HOAs) of the study participants.

	Normal control group (n = 38)	Atropine group (n = 40)	Orthokeratology group (n = 44)	Combined group (atropine + OrthoK) (n = 42)	*p* value	Age-adjusted *p* value^d^
High order irregularities (RMS)^a^	0.02 ± 0.01	0.02 ± 0.01	0.04 ± 0.02	0.06 ± 0.05	< 0.00001^c^	< 0.00001
Aberration coefficient (μm)^b^	0.38 ± 0.67	0.42 ± 0.68	1.88 ± 0.63	2.32 ± 1.21	< 0.00001^c^	< 0.00001

Parameters were shown by mean ± SD according to the character of each parameter. *p* < 0.05 was recognized as statistical difference.

^a^High order irregularities, presented with root mean square (RMS), calculated by Fourier analysis.

^b^Aberration coefficient, calculated by Zernike analysis.

^c^Kruskal–Wallis test statistics were used to test the group difference.

^d^Age-adjusted *p* value was analyzed by multivariate analysis of covariance through stepwise model selection of a multiple regression model that was adjusted by controlling the confounding variable of the age.

### Spatial Distribution of the first tear break-up

Newly categorized zones and representative TBUT-based color-coded maps of the 4 groups are demonstrated in Fig. 1. In the normal control group and the atropine group (Fig. 1b, c), the first break-up zones tended to be located at the outer half. On the other hand, in the groups wearing orthokeratology lenses (Fig. 1d, e), the first break-up zones were significantly more at the inner half (A + C 60.3%, B + D 39.7%; A + C 46.5%, B + D 53.5%; respectively; *p* = 0.04) under age-adjusted analysis.

**Figure 2:**
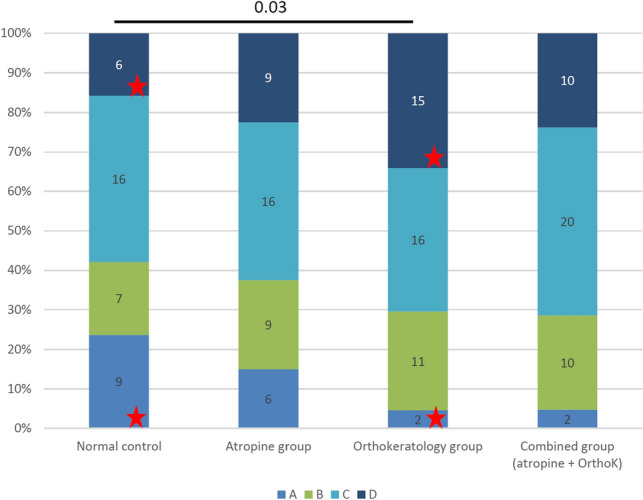
The spatial distribution and the corresponding percentage of the first tear break-up zone among the four groups. A significant distribution difference was detected between the normal control group and the orthokeratology group (*p* = 0.03), especially within zones A and D (*p* = 0.0126, red stars “ ”, by Chi-square test with Yate’s correction). Zones A to D was defined as the superior outer (more than 4 mm from the center), inner (less than 4 mm from the center) half, the inferior outer (more than 4 mm from the center) and inner (less than 4 mm from the center) half, respectively.

The distribution and the percentage of the first tear break-up zone among the 4 groups are shown in Fig. 2. A significant difference was observed between the normal control group and the orthokeratology group (*p* = 0.03) and the significant difference in distribution was attributed to the difference between zones A and D (*p* = 0.008).

**Figure 3:**
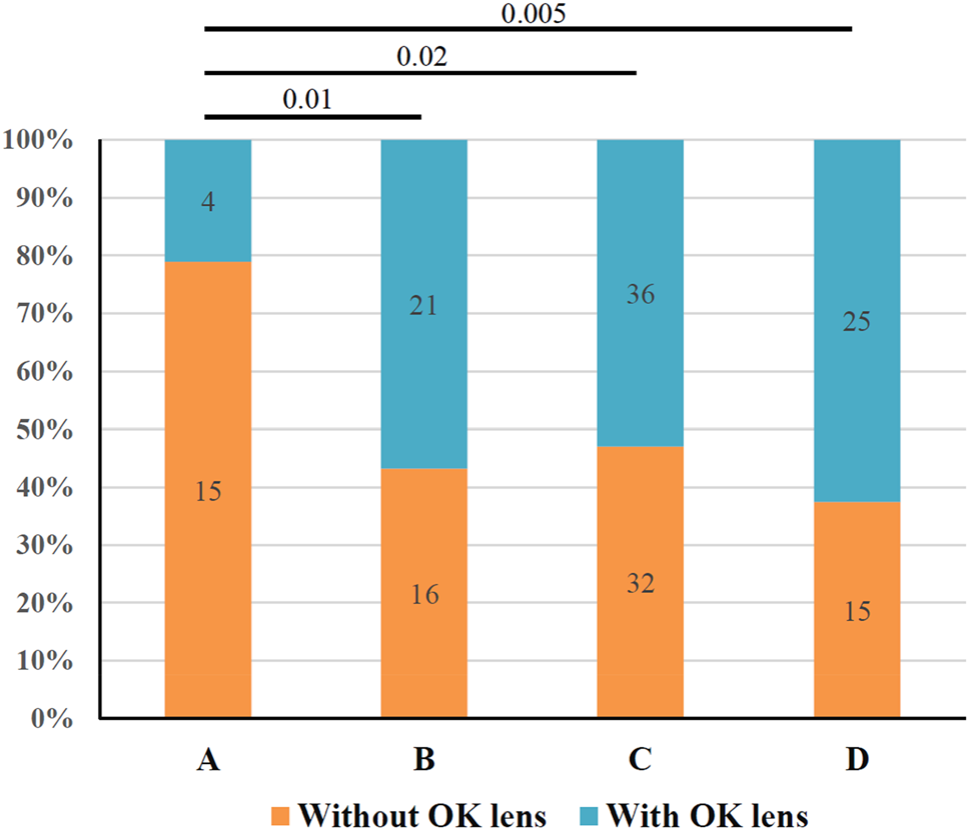
The spatial distribution and the corresponding percentage of the first tear break-up zone with and without orthokeratology wearing. Compared with patients without orthokeratology wearing, patients with orthokeratology wearing had significantly lower chance of the first tear break-up shown in zone A than in zone B–D (*p* = 0.01, 0.02, and 0.005 respectively by Chi-square test). Zones A to D was defined as the superior outer (more than 4 mm from the center), inner (less than 4 mm from the center) half, the inferior outer (more than 4 mm from the center) and inner (less than 4 mm from the center) half, respectively.

When the patients were grouped into orthokeratology and non-orthokeratology groups, the distribution of the first tear break-up zone revealed a significant difference between the groups (*p* = 0.02) (Fig. 3). When compared with patients from the non-orthokeratology group, patients wearing orthokeratology lenses had significantly lower proportion of the first tear break-up occurrence in zone A than in zones B to D (*p* = 0.01, 0.02, and 0.005, respectively).

### Influence of HOAs on spatial distribution of the first tear break-up

For elucidating the influence of HOAs on the newly categorized tear break-up zones, HOAs were compared according to the first tear break-up zones (Table 3). According to the result of Fourier analysis, children whose first tear break-ups were located at zone D had higher HOAs than children whose first tear break-ups were located at zones A and C (0.0439 ± 0.0416, 0.0276 ± 0.0209 and 0.0299 ± 0.016, respectively) while not statistically significance. However, based on Zernike analysis, children with first tear break-ups located at zone A to D showed a significant difference (0.68 ± 0.88, 1.37 ± 1.12, 1.22 ± 1.02, and 1.70 ± 1.24, respectively,* p* = 0.03). After conducting the Fisher's LSD post-hoc test between the aberration coefficient of the different zones, we found that children whose first tear break-ups were located at zone A had lower aberration coefficients than children whose first tear break-ups were located at zone D (*p* = 0.02).

**Table 3:** HOAs of the patients with the first tear break-up at different zones.

	A (n = 19)	B (n = 37)	C (n = 68)	D (n = 40)	*p* value
High order irregularities (RMS)^A^	0.0276 ± 0.0209	0.0367 ± 0.0346	0.0299 ± 0.016	0.0439 ± 0.0416	0.12
Aberration coefficient (μm)^B^	0.68 ± 0.88^a^	1.37 ± 1.12	1.22 ± 1.02	1.70 ± 1.24^a^	0.03

Parameters were shown by mean ± SD according to the character of each parameter. *p* < 0.05 was recognized as statistical difference. Analysis of variance (ANOVA) was used to test the differences between groups.

^A^High order irregularities of corneal surface, presented with root mean square (RMS), calculated by Fourier analysis.

^B^Aberration coefficient of corneal surface, calculated by Zernike analysis.

^a^Post hoc test revealed a significant difference of aberration coefficient between zone A and zone D, *p* = 0.02.

Moreover, we also detected that children with first tear break-ups located at the inner half (zone B and D) showed higher HOAs than children whose first tear break-ups were located at the outer half (zone A and C) (Fourier analysis: 0.0407 ± 0.0385 vs. 0.0294 ± 0.0169, *p* = 0.01; Zernike analysis: 1.55 ± 1.19, 1.11 ± 1.01, *p* = 0.01).

## Discussion

With a significant increase in the global prevalence of myopia, management and prevention of myopia-related ocular complications are considered matters of urgency. Moreover, the side effects associated with myopia treatments have raised considerable concerns^2^. The population of children treated with atropine and orthokeratology is increasing every year and the potential ocular surface damage should not be underestimated. Ocular surface damages caused by these treatments are generally initiated by tear film instability^12,17,19,30^. Therefore, thorough understanding of the tear film stability is highly important for pediatric myopia control. To the best of our knowledge, there has not been any research focusing on spatial disturbances of tear film in myopic children. Results of the present study revealed that myopic children treated with orthokeratology presented with higher HOAs and unique tear film spatial instability. The first tear film break-up zones in children treated with orthokeratology tended to be located at the inner half of the cornea, while the first tear film break-up zones in children without orthokeratology treatment were located at the outer half of the cornea. This phenomenon may be due to the fact that orthokeratology reduces the smoothness of the corneal surface and irregular corneal surface changes the spatial stability of the tear film.

Only a healthy ocular surface can provide a smooth optical surface for visual function and a normal tear film plays a dominant role in it^31^. Though tear film instability, which might be the initiator of severe ocular surface disease, has not yet been reported in myopic children under atropine control, it was recently announced and noticed as an unignorable risk in children under orthokeratology control^12,13,15,32^. Some researchers debated about the ocular surface alterations after overnight wearing of orthokeratology lenses. Na et al. proposed that overnight wearing of orthokeratology lenses may cause changes in the meibomian glands and tear film stability^15^. Wang et al.^12^ reported that overnight wearing of orthokeratology lenses could decrease TMH and TBUT but did not affect the function of the meibomian glands. However, Li et al.^32^ and Xie et al.^13^ pointed out that basal tear secretion and ocular surface inflammation were unaffected after orthokeratology treatment and there was only a short-term decrease in TBUT. They believed that the increase in tear evaporation may be related to the distribution of tear film caused by transient morphological changes in the tear film. In the present study, children under different myopia treatments showed no significant difference in TMH, TBUT, or ocular surface inflammation when compared with normal children (Table 1). The exact ocular surface damage in children with myopia control is reported less frequently than that in adults with myopia control. This may be due to more rapid recovery of the tear film stability and longer time needed to induce potential meibomian gland alteration in children. However, this interpretation could be dangerous, as these parameters may not represent the true tear film stability and may not reflect the potential ocular surface alteration. Hence, we aimed to discover other indicators for the early detection of tear film alteration.

Allergic conjunctivitis caused by atropine instillation has been reported^10^, but tear film alteration associated with atropine has not yet been investigated. Cai et al.^33^ suggested an effective control of epiphora with transcutaneous application of atropine gel, as atropine is an antagonist of muscarinic acetylcholine receptors, which might reduce the oversecretion of the transplanted submandibular gland for treating severe dry eye disease by modulating aquaporin-5 trafficking^34^. Therefore, topical instillation of atropine might also affect lacrimal gland secretion and alter the tear film on the ocular surface. In the present study, the amount of tear secretion and the severity of ocular surface inflammation revealed no significant differences between normal healthy children and myopic children receiving topical atropine treatment (Table 1). Moreover, the spatial stability of the tear film under atropine treatment was similar to that in normal children. In contrast to commercially available atropine gels (1% atropine sulfate gel), Cai et al.^33^ blended it with certain proprietary components to increase its transcutaneous permeability. Thus, the efficacy could last for 3–5 h. Myopic children in our study were treated with topical instillation of fortified low-concentration atropine (0.01–0.05%) a single drop per day. Besides, the eye drops were thought to leave the ocular surface rapidly due to their little volume and the pumping effect of the nasolacrimal system. These might be the possible reasons for almost no alterations in the tear film or the ocular surface after topical instillation of atropine.

On the other hand, we noticed that children treated with orthokeratology presented with significantly higher HOAs than the normal control group and the atropine group (Table 1). Moreover, the spatial stability of the tear film was altered in children treated with orthokeratology. Tear film plays an important role in obtaining good optical quality of the ocular surface, as it is the most anterior refractive surface of the eye and pathologic tear film irregularities can significantly disturb the light trail^35–37^. In patients treated with orthokeratology, the reshaped corneal surface could redistribute the tear film, resulting in tear break-up, which was thought to be located at an area with the largest corneal curvature^38,39^.

Bower et al.^40^ reported that the irregularity of the corneal surface would associate with dry eye disease due to the tear film instability. In dry eye disease, HOAs are increased and optical quality is degraded. HOAs were mostly analyzed by Zernike polynomials and Fourier harmonic analysis and were generally useful in dry eye disease to assess and monitor the tear film irregularities in patients during their disease courses^41–44^. To the best of our knowledge, there are no current researches focusing on HOAs and tear break-up zones in children under different myopia treatments. In the present study, we proposed a novel analysis of spatial distributions of the tear break-up in pediatric myopia and found different spatial distributions of the break-up points in children under different myopia treatments. The first tear break-up point in the normal control group was located more frequently at the superior outer zone and less frequently at the inferior inner zone than that in the orthokeratology group (Fig. 2). Moreover, when we grouped the patients into orthokeratology and non-orthokeratology groups, the difference became more obvious (Fig. 3).

When compared with children without orthokeratology treatment, children wearing orthokeratology lenses showed a significantly higher frequency of the first tear break-up point at the inner half (39.7% vs. 53.5%, *p* = 0.039). Difference in the spatial distribution of the first tear break-up might be related to the design of the orthokeratology lens. To obtain relative peripheral myopic defocus, the design of the orthokeratology lens adopts a smaller optic zone of less than 6 mm^45^ and the optic zone almost matches the inner zones (zones B and D) of the tear break-up area. In addition, when we categorized children according to the first tear break-up zone (zones A–D), children with the first tear break-up at zones B and D (inner half) had higher HOAs (Table 3). The contact of the optic zone with the corneal surface may alter the tear film distribution in the inner half of the cornea and may lead to a more rapid tear break-up at the inner half of the cornea.

Among children treated with orthokeratology, the measure time tended to be longer in children with higher HOAs despite a marginal significance (*p* = 0.068 by irregularity in Fourier analysis and *p* = 0.088 by aberration coefficient in Zernike analysis). This finding might be related to the low sensitivity of ocular discomfort induced by long-term use of contact lenses. Together with the definite increase in HOAs and the characteristic spatial distribution of the tear film, we came up with a hypothesis that orthokeratology wear-induced spatial instability might be associated with higher possibility of symptom-free dry eye disease, epithelial defect, and microbial keratitis due to microbial incursion. Further well-designed studies would be needed to prove this hypothesis.

The present study has some potential limitations. It was a cross-sectional study which could only reflect the tear performance at a single period of a day, so the daytime fluctuation and longitudinal change in the tear film performance could not be observed. A thorough observation of tear performance fluctuation in a day and a prospective cohort study initiating from the first atropine instillation or the first orthokeratology application needs to be designed for chronological observation of the changes in the tear film performance. However, the present study proposes an easy protocol during regular follow-ups that could offer a thorough understanding of the tear film performance and the ocular surface alteration after a certain period of treatment. In the present study, the subjective TMH measurement for determining the amount of tear secretion may have been affected by intra-observer difference. However, we believe that the difference was minimized, as the TMH for each participant was determined by a single masked interpreter and the mean value of 3 digital recordings was used for calculating the TMH in this study.

In summary, current myopia control modalities including atropine and orthokeratology have been proven to show good potential for retardation of myopia progression and no serious inevitable adverse effects have been reported under recommended dosage and instructions^46,47^. When compared with atropine, wearing orthokeratology lenses was associated with higher HOAs and might induce a characteristic spatial disturbance in the tear film stability. For pediatric patients under myopia control, especially for those under orthokeratology treatment, the spatial distribution of the tear film break-up might provide an easier, more rational and focused way of monitoring the pediatric tear film instability to better prevent any possible subsequent epithelial erosion, microbial inoculation, or development of microbial keratitis.
